# Let me know your name: a study of chigger mites (Acariformes: Trombiculidae) associated with the edible dormouse (*Glis glis*) in the Carpathian–Balkan distribution gradient

**DOI:** 10.1007/s10493-023-00824-0

**Published:** 2023-08-08

**Authors:** Paula Zajkowska, Tomasz Postawa, Joanna Mąkol

**Affiliations:** 1 Department of Invertebrate Systematics and Ecology, Institute of Environmental Biology, Wrocław University of Environmental and Life Sciences, Kożuchowska Str. 5b, 51-631 Wrocław, Poland; 2 Landscape Ecology Team, Institute of Systematics and Evolution of Animals, Polish Academy of Sciences, Sławkowska Str. 17, 31‐016 Kraków, Poland

**Keywords:** Chiggers, Europe, Host, Larvae, Integrative taxonomy, Hidden diversity

## Abstract

Trombiculid mites were collected from the edible dormouse (*Glis glis*) within the Carpathian–Balkan distribution gradient of host species. Representatives of five genera (*Leptotrombidium*, *Neotrombicula*, *Brunehaldia*, *Hirsutiella*, *Schoutedenichia*) and 10 species of chiggers were discovered in the material, based on morphological and/or molecular data. *Brunehaldia*, new to the fauna of Greece, was recorded for the first time from the edible dormouse. *Neotrombicula talmiensis* was new to the fauna of Greece and *Neotrombicula vulgaris* was new to the fauna of North Macedonia. Successful amplification and sequencing of COI was carried out in relation to three genera and six species. The intraspecific variation of taxa hitherto distinguished based on morphological criteria was juxtaposed with molecular data, using the distance method and the phylogenetic approach. The molecular methods indicated wider than hitherto recognized, intraspecific morphological variation for *Leptotrombidium europaeum* and *N. talmiensis*. On the other hand, an inference limited to morphology proved to be insufficient for species delineation, which was confirmed by the relatively low identity (%) of examined COI sequences as well as the size of inter-/intraspecific K2P distance threshold. Our study provides support for integrative taxonomy that combines different sources of evidence and contributes to recognition of the scope of intraspecific variation. The high degree of hidden diversity revealed with the application of molecular tools, votes for a careful approach to the identification of chiggers. The confirmed cases of co-invasion, including the representatives of various genera (*Leptotrombidium* and *Neotrombicula*, *Brunehaldia* and *Neotrombicula*, *Neotrombicula* and *Schoutedenichia*, *Hirsutiella* and *Schoutedenichia*) additionally support the need to include all larvae found on a given host specimen in the identification process.

**Supplementary Information:**

The online version contains supplementary material available at 10.1007/s10493-023-00824-0.

## Introduction

Trombiculidae (sensu Kudryashova 1998; Shatrov and Kudryashova 2008) comprise Trombiculinae, Leeuwenhoekiinae, Gahrliepiinae and Apoloniinae. According to the most recent inventory (Nielsen et al. 2021), 188 genera and 3013 species have been hitherto described for the family (excl. ca. 20 species described in 2021/2022), and around 200 species have been recorded from the western Palearctic (Kudryashova 1998; Stekolnikov and Daniel 2012). The unstable taxonomy of Trombiculidae is shown by the about 5500 synonymies, redescriptions, and new combinations of species names recorded between 1921 and 2021, and summarized by Nielsen et al. (2021).

For over 260 years the specific affiliation of chiggers has been ascertained based on morphology. However, a scarce knowledge of intraspecific variation, as well as species diagnosing based on limited material, without critically addressing all published characteristics of a species, constitute common problems in chigger taxonomy. An additional difficulty concerns the keys for species identification that often refer to the character states of specimens collected from selected areas within the geographic limits of the species, and not to the specimens originating from the entire distribution range. In view of the above but also in view of the great scarcity of other evidence that would support the correct identification of chiggers, any association of a name with a set of morphological—both qualitative and quantitative—characters, is burdened with a risk of misidentification. An additional obstacle lies in the frequently applied ‘serial’ identifications that consider hundreds of specimens assigned to the same species, without paying enough attention to the relatively common phenomenon of co-invasion. The latter may constitute a real pitfall, especially in the case of similar species.

The molecular techniques (with special reference to DNA sequencing) that have been increasingly applied in chigger taxonomy in recent years (e.g., Shao et al. 2006; Moniuszko et al. 2015; Antonovskaia 2018; Jacinavicius et al. 2018) contribute to the verification of species statuses. Their role in answering the question of what is behind a name cannot be overestimated, although for obvious reasons it seems to be a long-term task. In hitherto research, the most often used markers for taxonomic identification of chiggers and inference at various levels of relatedness were the nucleotide sequences of the mitochondrial gene coding for cytochrome oxidase subunit I (COI) (Young et al. 2012; Moniuszko et al. 2017, 2018; Zajkowska and Mąkol 2022), the small ribosomal subunit 18S rRNA (18S) gene (Pepato et al. 2010; Mendoza-Roldan et al. 2017; Bassini-Silva et al. 2018), and—to a lesser extent—the large ribosomal subunit 28S rRNA (Pepato et al. 2010; Klimov et al. 2018). Still limited feedback, compared to some other terrestrial Parasitengona families, is due to the low success rate recorded in the analyzes, as evidenced by the small number of topic-related publications and the small number of sequences assigned to species (> 330 COI sequences; 14 18S rRNA sequences; four 28S rRNA sequences (GenBank, accessed 26 January 2023).

Larvae of Trombiculidae are considered as habitat-specific rather than host-specific ectoparasites (Goff 1979); however, small mammals, such as rodents, bats, and insectivores, are among the most frequent hosts for chiggers (Shatrov and Kudryashova 2008). Here we address the question of species diversity of Trombiculidae infesting the edible dormouse, *Glis glis* (L.) (Rodentia: Gliridae). The species is considered arboreal (Cornis et al. 2017); however, some literature data (e.g., Vikyrchak and Ploshchansky 2020) point to its tendency to occupy subterranean habitats.

To date, 14 chigger species, identified based on morphology, have been recorded from this host. The inventory of records, provided by Kirillov et al. (2022), comprising 12 species—*Ascoschoengastia latyshevi* (Schluger), *Hirsutiella zachvatkini* (Schluger), *Leptotrombidium europaeum* (Daniel et Brelih), *Leptotrombidium silvaticum* Hushcha et Schluger, *Miyatrombicula muris* (Oudemans), *Neotrombicula austriaca* Kepka, *Neotrombicula inopinata* (Oudemans), *Neotrombicula japonica* (Tanaka, Kaiwa, Teramura et Kagaya), *Neotrombicula nagayoi* (Sasa, Hayashi, Sato, Miura et Asahina), *Neotrombicula vernalis* (Willmann), *Neotrombicula vulgaris* (Schluger), and *Schoutedenichia* sp.—should be completed with two more species, recorded by Kepka (1964) and Mulyarskaya (1965): *Neotrombicula autumnalis* Shaw and *Neotrombicula talmiensis* (Schluger). Of those, *N. autumnalis* has been known as widely distributed within the geographic range of *G. glis*; however, its status should be verified as likely to represent several taxa. The occurrence of *N. vernalis* on *G. glis* reported by Kirillov et al. (2022, referring to Kepka 1964) should be confirmed due to the inconsistency of data provided in the original publication.

The aim of the present study was to trace the species composition of ectoparasitic chiggers associated with *G. glis*, within the Carpathian–Balkan distribution gradient of the host species. The sampling was focused on subterranean habitats of potential occurrence of the host species. We have also attempted to verify the identity of trombiculid species with the application of molecular techniques to check the congruence between various input data used for species identification.

## Material and methods

### Sampling

**Fig. 1:**
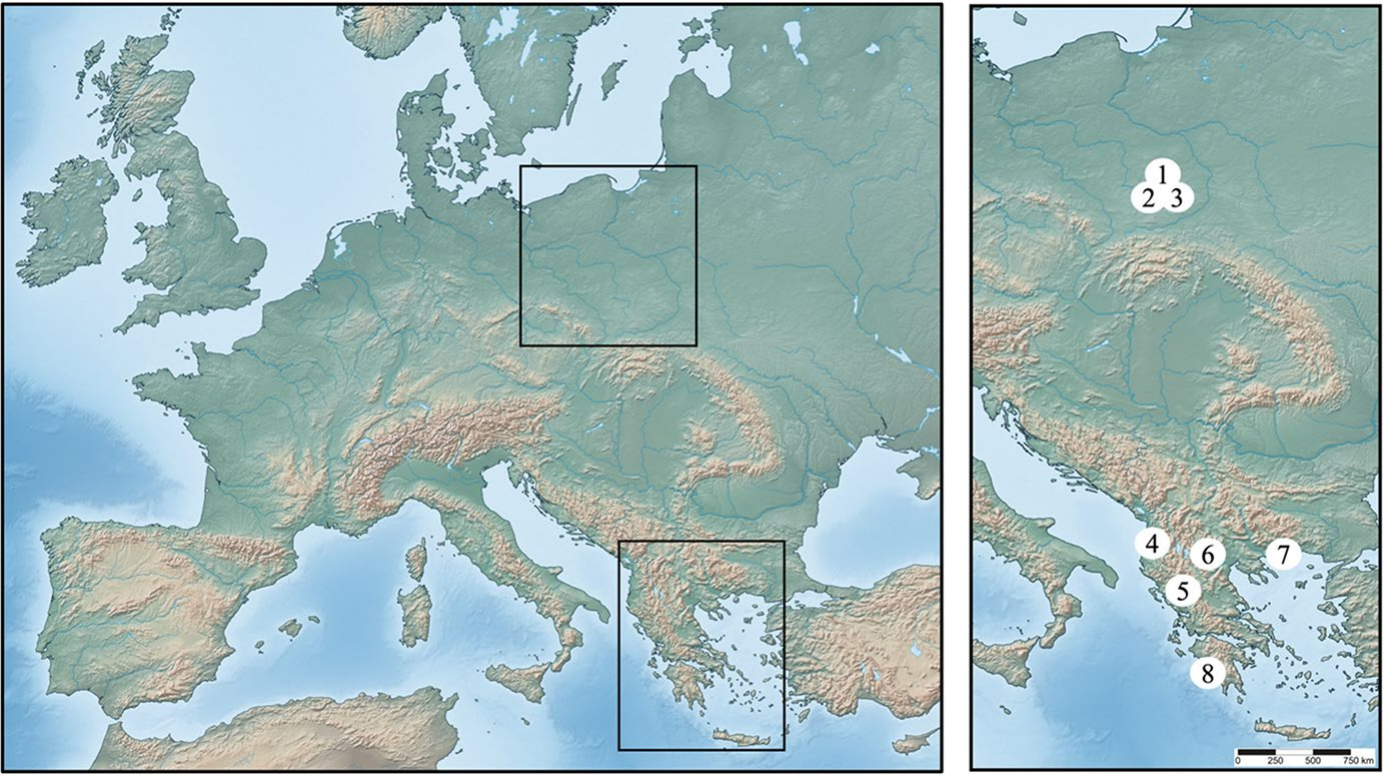
Collecting sites of *Glis glis* infested with chiggers: Poland (Kraków-Częstochowa Upland): [1] Chuda Cave (50° 40′ N, 19° 25′ E), [2] Wiercica Cave (50° 40′ N, 19° 24′ E), [3] Wilcza Góra Cave (50° 40′ N, 19° 26′ E); Albania—concrete military tunnels: [4] Perlat (41° 43′ N, 19° 59′ E) and [5] Langarica Canyon (40° 14′ N, 20° 25′ E); North Macedonia: [6] caves near Kamienica valley Dragonzhel, Kavadarci (41° 18′ N, 22° 02′ E); Greece: [7] Thasos Island, Drakotrypa Spilaio Cave (40° 43′ N, 24° 43′ E) and [8] Pylos Island, drift (36° 56′ N, 21° 44′ E)

Trombiculid larvae were sampled from edible dormouse in 2020–2022, at underground shelters: bunkers (concrete military tunnels) and caves, in Poland (permissions: WPN.6205.31.2020.MM, WPN.6205.15.2022.MM), Albania, North Macedonia, and Greece (Fig. 1). Rodents were collected using Sherman traps (23 × 8 × 9 cm) in Poland, or caught directly by hand in the other countries. The entire body surface of each potential host specimen was screened for larvae. Parasitized individuals were temporarily transferred to a cotton bag, to avoid cross-contamination with ectoparasitic larvae. Chiggers were removed from the hosts using smooth forceps and transferred directly to 96% EtOH. Afterwards, all rodents were released unharmed in their natural habitat.

### DNA extraction, amplification, and sequencing

The genomic DNA was extracted from the entire (non-punctured) larval specimens; the exoskeletons that remained after extraction were mounted on microscope slides for purpose of morphological examination. Polymerase chain reaction, and selection of programs used for sequence analyses follows Zajkowska and Mąkol (2022) and Mironov et al. (2012). The COI gene was PCR amplified by primers LCO1490/HCO2198 (Folmer et al. 1994), bcdF01/bcdR04 or bcdF04/bcdR04 (Dabert et al. 2010), and the nuclear 28S rDNA, including the D2 region, was amplified with primers 28SF0001/28SR0990 (Mironov et al. 2012). In selecting the subset of samples intended for 28S rDNA sequencing, the quality of agarose gel electrophoresis of PCR products was assessed and samples that suggested the separate species affiliation after COI analyses were forwarded for sequencing targeting at 28S rDNA gene. DNA sequencing of PCR products on both strands was performed by Genomed (Poland).

Contigs were assembled in Geneious v.9.1.8 (https://www.geneious.com). Sequence similarity search against sequences stored in GenBank was done using BLASTn search implemented in Geneious. The single haplotype sequences obtained in this study were deposited in the GenBank under the following accession numbers: OQ924402–OQ924413 (COI) and OQ925889–OQ925891 (28S).

### Alignment

The multiple sequence alignment consisting of COI and 28S sequences was produced in Geneious using the MAFFT algorithm (gap opening penalty: 1.53). The published sequences of Trombiculidae and of outgroup taxa (Bdellidae spp.) (Table S1), retrieved from GenBank, served for comparison.

### Species delimitation

For species delimitation based on COI sequences, a phylogenetic method – generalized mixed Yule coalescent (GMYC; Fujisawa and Barraclough 2013) – was applied, as well as two distance matrix methods – automatic barcode gap discovery (ABGD; Puillandre et al. 2011) and ‘assemble species by automatic partitioning’ (ASAP; Puillandre et al. 2021). The GMYC adopts a likelihood approach to analyze the timing of branching events, seeking for significant switches between a Yule (interspecific) and a coalescent (intraspecific) branching structure (Vences et al. 2021). The maximum likelihood ultra-metric tree was inferred in BEAST v.2.6.7 (Bouckaert et al. 2019). The GMYC analysis was done in R software (R Core Team 2016) using the packages *ape*, *paran*, *rncl* and *splits*.

The web-based program interfaces were used for ABGD (https://bioinfo.mnhn.fr/abi/public/abgd/), which calculates the pairwise distance based on the barcode gap, and for ASAP (https://bioinfo.mnhn.fr/abi/public/asap), which uses the hierarchical clustering algorithm based on pairwise genetic distances. Genetic distance between sequences (alignments created from COI sequences obtained during this study and those retrieved from GenBank but also the subsets of COI and 28S sequences obtained during the present study) was estimated with Kimura-2 parameter (K2P) substitution model (Kimura 1980) in MegaX (Kumar et al. 2018).

To determine the species affiliation of chiggers, the results of delimitation were compared with the results of morphological analysis.

### Morphological analysis

The larvae (the entire specimens as well as exoskeletons that remained after DNA extraction) were mounted on microscope slides in Faure’s fluid (Walter and Krantz 2009). The morphological examination of larvae, including measurements, was carried out under a Nikon Eclipse E600 compound microscope, equipped with differential interference contrast (DIC) and DS-Fi1 camera system, using the NIS-Elements BR software.

In morphological identification of specimens to genus and species level we referred to identification keys as well as to original descriptions and redescriptions (Vercammen-Grandjean and Langston 1976; Kolebinova 1992; Kudryashova 1998; Fernandes and Kulkarni 2003; Stekolnikov and Daniel 2012; Stekolnikov 2013, 2018). The morphological terminology and abbreviations appearing in the text follow Stekolnikov (2013). Additionally, the LV denotes larva/e. The slide-mounted material is stored at the Department of Invertebrate Systematics and Ecology, Wrocław University of Environmental and Life Sciences.

The following sequence of issues was adopted for species accounts: current name, synonymy, material examined (followed by the total number of larvae/larval exoskeletons subjected to morphological analyses and the number of sequences, if obtained; numbers in square parentheses refer to the localities in Fig. 1), diagnosis (incl. verified diagnosis), distribution and hosts, remarks (optional).

### Phylogenetic analysis

Models of nucleotide substitution (GTR + G + I) were evaluated for COI alignment (own sequences and sequences retrieved from GenBank) using MegaX based on Bayesian information criterion (BIC) and Akaike’s information criterion (AIC). The maximum likelihood (ML) phylogenetic tree was created with MegaX (Kumar et al. 2018); support values for ML branches were generated with the bootstrap method, with 1000 replicates. The phylogenetic tree visualization was made using iTOL (https://itol.embl.de).

## Results

**Fig. 2:**
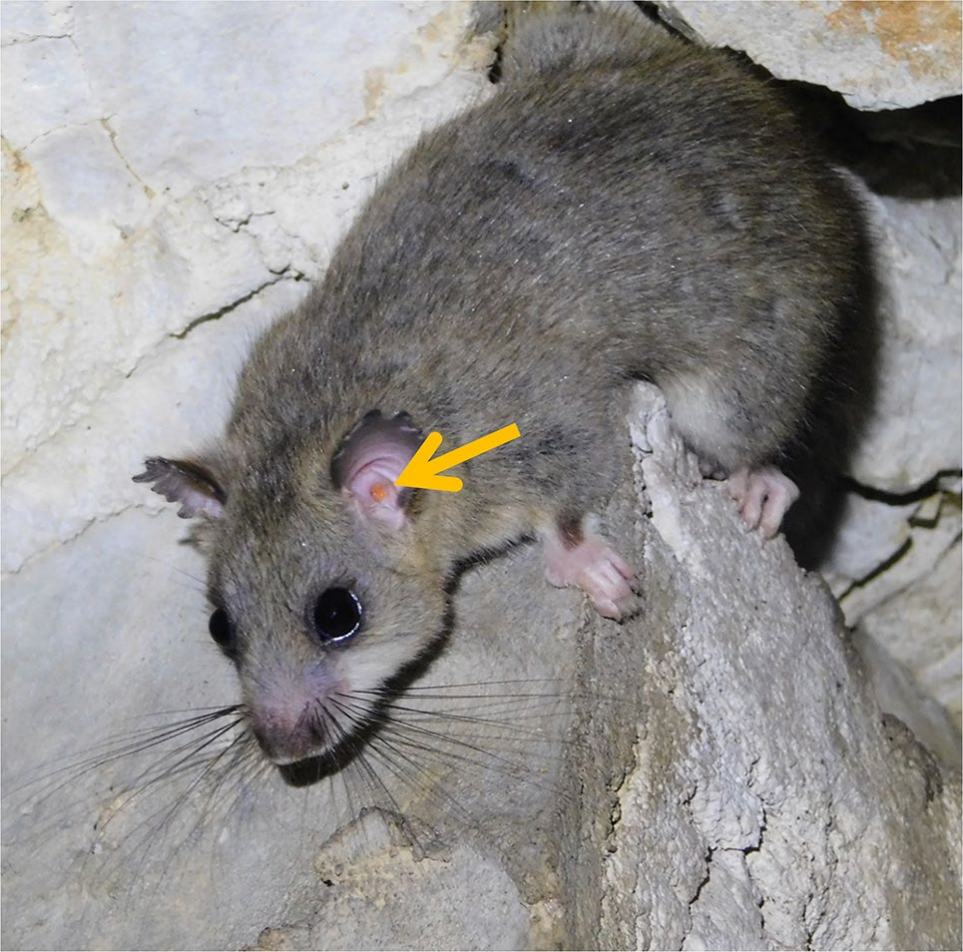
The edible dormouse (*Glis glis*) infested with chiggers (the arrow points to larvae in the host’s ear), in subterranean habitat, typical of Karst areas

In total, 94 chiggers were collected from 15 specimens of *G. glis*, at eight localities in Poland, Albania, North Macedonia, and Greece (Fig. 1). Larvae were found exclusively within hosts’ ears (Fig. 2). Fifty-two larvae were selected for further, molecular and/or morphological analyses.

### Sequence performance

Of 46 specimens intended for molecular studies, we received 25 COI sequences, which accounted for an overall barcoding success of 54%. We also obtained seven sequences of 28S rDNA. Twelve COI sequences and three 28S sequences were represented by unique haplotypes. Due to the low number of 28S sequences obtained, these sequences could be used for comparative purposes to a limited extent.

The value of genetic divergence between 12 COI single haplotype sequences (Table S2), that produced 573 bp sequence alignment, suggested the presence of three genera in the part of the material examined with the application of molecular tools. The genera were supported by BLASTn comparison, allowing for assignment of the sequences to *Leptotrombidium*, *Neotrombicula* and *Schoutedenichia*. The genetic distance was 35.5–37.5% between *Leptotrombidium* and *Neotrombicula*, 40.5–43.6% between *Leptotrombidium* and *Schoutedenichia*, and 28.4–32.1% between *Neotrombicula* and *Schoutedenichia*.

### Molecular species delimitation

The species delimitation was carried out on two subsets of sequences assigned to two genera, *Neotrombicula* and *Leptotrombidium*, based on sequence similarity search.

**Fig. 3:**
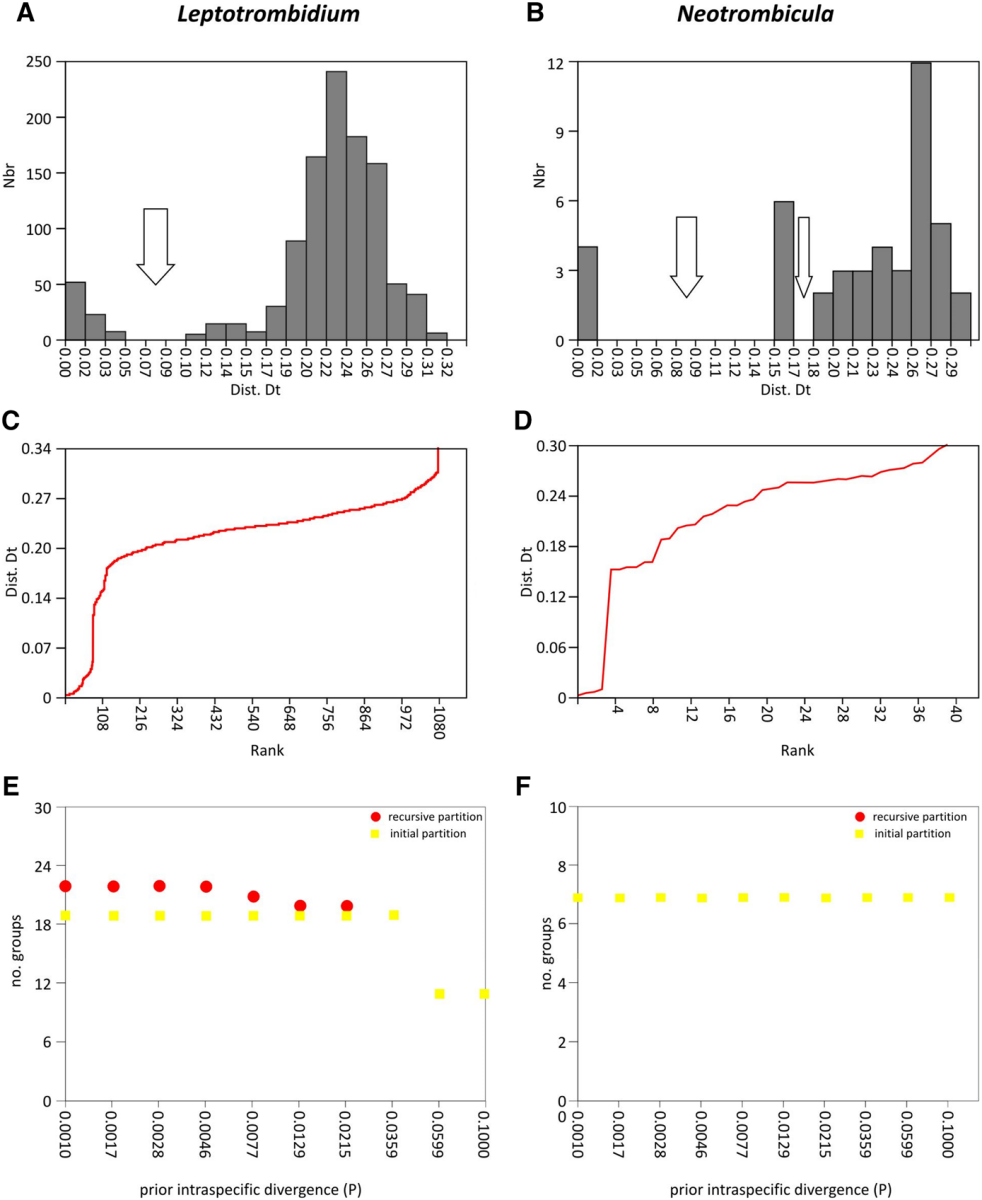
Graphical output of automatic barcode gap discovery (ABGD) analysis of sequences of *Leptotrombidium* spp. **A**, **C**, **E** and *Neotrombicula* spp. **B**, **D**, **F**. A, B—Distribution of pairwise distances between 47 COI sequences (434 bp) and between 10 COI sequences (439 bp). C, D—Ranked pairwise (K2P) distances. E, F – number of initial (E, F) and recursive (E) partitions

*Leptotrombidium* alignment (434 bp) composed of 47 single haplotype sequences, including five obtained during the present study and 42 retrieved from GenBank (Table S1), pointed to the occurrence 19 operational taxonomic units (OTU) in ASAP (asap-score = 3.5) and GMYC, and of 19 OTU and 21 (or 22) OTU in ABGD, at initial partition and at recovery partition, respectively. The sequences (n = 5) obtained from chiggers collected from *G. glis* formed two separate OTUs, with 7.9% distance threshold value, irrespective of the method of delimitation applied. In ASAP and ABGD the barcode gap within *Leptotrombidium* revealed a 5–10% p-distance between putative species (Fig. 3).

*Neotrombicula* alignment (439 bp) composed of 10 unique haplotype sequences, including six obtained during the present study and four retrieved from GenBank (Table S1), indicated the occurrence of seven putative species (already at initial partition). Of those, three putative species were represented by larvae collected from *G. glis*. The results of delimitation were consistent for all three methods, ABGD, ASAP (with asap-score = 1.5), and GMYC. In the case of ABGD and ASAP the same distance threshold value, 8.1%, was calculated. In ASAP and ABGD the barcode gap within *Neotrombicula* revealed 2–15% and 17–18% p-distance, respectively, between groups representing putative species (Fig. 3).

### Species account and taxonomy

At preliminary, morphology-based inference at the level of genus we used four alcohol-preserved specimens and 32 exoskeletons recovered after DNA extraction. In the case of nine slides, their quality did not allow the in-depth morphological analysis, thus four alcohol-preserved specimens and 23 exoskeletons that remained after DNA extraction served for morphology-based, species-level inference.

We confirmed the presence of three genera preliminarily distinguished based on molecular analyses. For the two other genera, in the lack of legible sequences, the morphology served as the only source of identification. Finally, the members of the following genera were recognized in the examined material: *Leptotrombidium*, *Neotrombicula*, *Schoutedenichia*, *Hirsutiella* and *Brunehaldia*.

### *Leptotrombidium*

#### *Leptotrombidium europaeum* (Daniel et Brelih)

*Material examined.* 12 LV (one entire specimen and seven exoskeletons mounted on slides; 11 COI sequences): [1] Poland, Chuda Cave, 19 June 2020, leg. TP, 3 LV (two exoskeletons; three COI sequences/one haplotype [OQ924407]); [2] Poland, Wiercica Cave, 20 May 2020, leg. TP, 2 LV (one entire specimen and one exoskeleton; one COI sequence/one haplotype [OQ924402]) and 16 June 2020, leg. TP, 5 LV (two exoskeletons; five COI sequences/two haplotypes [OQ924403 and OQ924406]); [3] Poland, Wilcza Góra Cave, 15 June 2022, leg. PZ, 2 LV (two exoskeletons; two COI sequences). For metric and meristic data see Table 1.

**Table 1:** Morphological data on *Leptotrombidium europaeum* and *Leptotrombidium* sp. A

Trait	*Leptotrombidium europaeum*					*Leptotrombidium* sp. A	
	Daniel and Brelih 1959^a^ Slovenia (n = 11)	Vercammen-Grandjean and Langston 1976^c^ (n = 10)	Stekolnikov 2004 Russia (n = 65)	Stekolnikov and Daniel 2012^d^ Turkey (n = 39)	This study^e^ Poland (n = 8)	This study Albania (n = 2)	
						a	b
SB	32–43	33–41	33–44	34–43	35–42	40	38
AW	73–84	75–83	72–88	72–85	71–90	86	77
PW	85–95	88–92	81–100	81–97	75–101	**102**	88
AP	25–31	24–29	24–34	23–32	18–28	27	26
ASB	29–35	30–34	26–37	27–35	23–30	30	26
PSB	17–21	18–21	16–23	16–21	13–20	20	17
P-PL	–	–	14–23	15–23	10–23	17	18
AM	60–70	60–66	49–68	50–64	52–63	57	57
AL	36–48	40–47	35–47	36–46	34–41	43	42
PL	60–71	60–70	52–72	55–70	56–66	59	61
S	66–78	78–84	65–86	68–82	55–67	–	–
SD	48–58	49–53	44–60	44–55	39–50	50	43
Leg I	268^b^	268–286	297–351	297–331	224–277	268	259
Leg II	236^b^	231–249	259–317	261–297	216–248	241	256
Leg III	284^b^	274–303	297–356	297–329	250–283	305	260
Ip	788^b^	776–820	855–1017	855–941	690–805	814	775
D_min_	–	–	40–54	40–52	31–44	49	49
D_max_	–	–	52–69	52–69	53–64	59	58
V_min_	–	–	36–56	30–34	26–39	26	28
V_max_	–	–	36–56	46–48	30–46	44	47
H	68^b^	63–71	54–73	53–72	51–66	59	62
DS	–	–	35–51	35–47	37–50	46	**58**
VS	–	–	29–51	29–43	24–35	28	26
NDV	–	–	69–102	69–89	70–76	74	85

Values of metric traits of *Leptotrombidium* sp. A departing from the scope of variation of *L. europaeum* are indicated in bold. Trait codes (first column) explained in Stekolnikov (2013)

^a^With the following exception: S (n = 9)

^b^Data for holotype (after Vercammen-Grandjean and Langston 1976)

^c^No data on the country of origin of the material

^d^With the following exceptions: S (n = 10); V_min-max_ (n = 2)

^e^With the following exceptions: AM and PW (n = 7); S, D_min-max_, V_min-max_, H and NDV (n = 5); VS and DS (n = 4)

*Diagnosis* (after Vercammen-Grandjean and Langston 1976; Stekolnikov and Daniel 2012; Stekolnikov 2013). SIF = 7B-B-3-2111.0000; fPp = N/N/BNN; fCx = 1.1.1; fSt = 2.2; fSc: PL > AM > AL; Ip = 7761017; fD = 2H-(8–15)-(8–12)-(711)-(5–11)-(…), basic formula 2H-10-8-8-6-4-2-2; DS = 35–51; VS = 29–51; NDV = 69–102.

#### Distribution and hosts

Hitherto records originate from Albania, Austria, Azerbaijan, Belarus, Bulgaria, Czechia, Estonia, France, Latvia, Lithuania, North Macedonia, Moldova, Montenegro, Poland, Romania, Russia, Serbia (Kosovo), Slovakia, Slovenia, Spain, Turkey and Ukraine; species of a wide range of insectivorous and rodent hosts (Moniuszko and Mąkol 2014, and references therein).

#### Remarks

A variable number of setae attributed to subsequent rows on the opisthosoma dorsum—i.e., fD = 2H-8-8-6-(…); 2H-9-8-6-(…); 2H-10-8-8-(…)—was observed in specimens that revealed significant similarity or identity (97.4–100%) of sequences, including the states 2H-9-8-6-(…) and 2H-10-8-8-(…) observed in case of two specimens that produced identical sequences (0% K2P distance). In case of the first two rows (C and D), the number of setae was consistent with the data provided in the diagnosis; in case of the third row (E), three out of eight specimens had six setae. Moreover, in five specimens lower values of Ip (690, 723, 737, 759, 762) were observed, compared to data provided in the diagnosis of *L. europaeum*. Yet another larva, with Ip = 805 falling within the variability range known for the species (Ip = 776–1017), shared 98.7 and 97.3% identity with larvae, for which the respective values of Ip, 690 and 737, went beyond those hitherto recorded for *L. europaeum*. Some specimens were also characterized by shorter setae AM, AL, PL. The shape of the scutum and of dorsal and ventral setae on idiosoma was consistent with one provided in the original description. Due to the scale of differences, and at a high level of identity of COI sequences of examined specimens, the observed deviations from the range of metric traits known for *L. europaeum*, are regarded here as manifesting the intraspecific variation. The following data should be incorporated in the modified diagnosis: (…) Ip = 6901017; fD = 2H-(8–15)-(8–12)-(611)-(5–11)-(…).

Based on discriminant analysis, Stekolnikov (2004) described two species—*Leptotrombidium alanicum* Stekolnikov and *Leptotrombidium paradux* syn. *montanum* Vercammen-Grandjean and Langston—morphologically similar to *L. europaeum*, noting that high geographical variation was the reason for poor species differentiation at quite distinct differences in sympatrically occurring specimens. Some specimens from the sample analyzed here fell within the range of *L. alanicum*. The status of *L. alanicum* and *L. paradux*, so close to *L. europaeum* (e.g.), also requires verification in terms of possible synonymy.

The set of morphological character states of only one of the specimens preliminarily assigned here to *L. europaeum* was fully compliant with *L. europaeum* according to the keys of Kudryashova (1998) and Stekolnikov (2013). However, in the key provided by Stekolnikov (2013) the data contained in the modified diagnosis (Stekolnikov and Daniel 2012) were not included. The identical or highly similar COI sequences obtained from specimens examined in the present study vote for their common specific affiliation.

Eleven COI sequences represented four haplotypes. Genetic distance between four haplotypes of *L. europaeum* (alignment 573 bp) varied between 0.9 and 2.9% (Table S2). All four sequences were classified within one OTU.

The measure of the genetic distance points to the common species identity of the examined specimens and also confirms the relatively wide variation of morphological traits in *L. europaeum*.

#### *Leptotrombidium* sp. A

*Material examined*. 2 LV (one entire specimen and one exoskeleton mounted on slides; one COI sequence [OQ924404], one 28S sequence [OQ925889]): [4] Albania, Perlat, 10 Oct. 2020, leg. TP. For metric and meristic data see Table 1.

*Remarks*. Both specimens revealed the similarity to *L. europaeum*. The values of PW and DS only slightly departed from those known for *L. europaeum* (Table 1). Both larvae of *Leptotrombidium* sp. A were collected from the same host specimen. Based on the key by Stekolnikov (2013) one specimen [OQ924404] fits *L. europaeum*, and the other one [specimen 8203/1] fits *L. europaeum*/*L. alanicum*. The specimens differ from each other in the number of setae in the first two rows on opisthosoma dorsum: 2H-14-9-(…) and 2H-10-10-(…), but the observed character states still fit the diagnosis of *L. europaeum* provided by Stekolnikov and Daniel (2012).

Genetic distance between the COI sequence obtained from OQ924404 and four sequences of *L. europaeum* (alignment 573 bp) was 10.1–11.5% (Table S2). Species delimitation assigned OQ924407 to separate OTU.

The ultimate decision concerning the affiliation of specimens requires further study of more extensive material, aiming at in-depth recognition of intraspecific genetic variation in Trombiculidae and translating into the scope of variation of morphological traits.

### *Neotrombicula*

#### *Neotrombicula talmiensis* (Schluger)

*Material examined*. 12 LV (nine exoskeletons mounted on slides; nine COI sequences/three haplotypes [OQ924410; OQ924411; OQ924413]; five 28S sequences/one haplotype [OQ925891]): [8] Greece, Pylos, 9 Oct. 2021, leg. TP. For metric and meristic data see Table 2.

**Table 2:** Morphological data on *Neotrombicula* spp.

Trait	*N. talmiensis*				*N. vulgaris*			*Neotrombicula* sp. A	
	Kolebinova 1992 Bulgaria^a^	Stekolnikov et al. 2014 Italy (n = 2)		This study Greece (n = 8)^b^	Stekolnikov 1999^c^ RUS, GEO, BUL, TURN (n = 76)	Moniuszko et al. 2017 Poland (n = 19)	This study North Macedonia (n = 1)	This study Greece (n = 2)	
		a	b					a	b
SB	33–45	35	32	29–33	31–40	30–36	34	31	32
AW	73–81	78	75	67–79	72–86	70–82	76	68	71
PW	88–107	92	90	85–92	90–104	88–98	99	**84**	85
AP	23–34	24	25	24–29	25–34	29–33	26	**20**	24
ASB	28–38	34	34	24–29	26–32	28–38	22	**22**	27
PSB	25–34	22	23	21–26	25–32	26–33	28	**19**	22
P-PL	–	27	29	24–27	21–31	23–29	26	**22**	24
AM	45–58	–	54	39–47	40–57	50–56	–	44	44
AL	40–48	43	41	33–48	38–49	41–51	37	40	42
PL	58–77	65	68	57–66	47–68	56–65	65	**56**	61
S	73–88	–	–	63; 69	68–90	81–91	–	64	66
SD	53–72	56	58	47–55	54–63	55–67	50	41	49
Leg I	253–290	275	265	245–290	241–326	281–318	246	232	246
Leg II	240–277	248	238	221–264	220–281	259–288	226	216	222
Leg III	280–328	295	288	245–304	256–324	285–326	267	247	265
Ip	773–888	819	790	716–858	725–916	839–922	740	**695**	732
D_min_	48/40	49	50	38–43	37–49	41–46	47	40	38
D_max_	73/59	59	65	50–53	54–63	52–57	54	47	56
V_min_	27/43	31	34	23–27	25–41	29–34	39	**21**	23
V_max_	45/63	56	65	32–36	38–61	44–52	54	32	39
H	55–75	67	70	53–63	47–67	59–66	60	**50**	59
DS	34	30	30	30–38	43–63	42–48	–	–	–
VS	31	33	30	20–28	24–37	28–38	–	–	–
NDV	65	63	60	50–60	72–95	66–76	73	50	54

Values of metric traits of *Neotrombicula* sp. A departing from the scope of variation of *N. talmiensis* are indicated in bold. Trait codes (first column) explained in Stekolnikov (2013)

^a^No data on the sample size

^b^With the following exceptions: AM (n = 4); AL (n = 7); S (n = 2); DS and VS (n = 6)

^c^Material originating from Russia, Georgia, Bulgaria, Turkmenistan

*Diagnosis* (after Stekolnikov et al. 2019): SIF = 7BS-B-3-3111.1000; fPp = B/B/N(B)BB; fsp = 7.7.7; fCx = 1.1.1; fSt = 2.2; fSc: PL > AM ≥ AL; fD = 2H-8-6-6-4-6-2, 2H-6-6-6-4-4-2; DS = 30–36; VS = 27–36; NDV = 59–70; Ip = 835–929; eyes 2 + 2; f_1_ anterior to S_1_; f_2_ posterior to S_2_.

*Distribution and hosts*. Hitherto records originate from Albania, Armenia, Azerbaijan, Bulgaria, China, Czechia, Georgia, Hungary, Iran, Italy, Kazakhstan, Kyrgyzstan, Moldova, Poland, Romania, Russia, Slovakia, South Korea, Turkmenistan, Ukraine; hosts: mammals—rodents, soricomorphs, eulipotyphlas (formerly within insectivores), lagomorphs, bats, carnivores—and birds (Moniuszko and Mąkol 2014; Shamsi et al. 2020, and references therein).

#### Remarks

Species new to the fauna of Greece. Larvae originate from three specimens of *G*. *glis* collected at one locality. All specimens share the palpal chaetotaxy formula fPp = B/B/BBB. The range of metric and meristic traits, except for slightly departing values (recorded in each case for one specimen), falls within or overlaps with the published data on *N. talmiensis* (Kolebinova 1992; Stekolnikov et al. 2014). The lower number of setae on the dorsal and ventral side of opisthosoma (NDV = 50, n = 1), higher maximum number of dorsal setae (DS = 38) and lower minimum number of ventral setae (VS = 20, n = 1), as well as shorter legs (Ip = 716, n = 1), at a high level of identity of COI sequences examined, should be incorporated in the modified diagnosis of the species.

The genetic distance between three single haplotype sequences of *N. talmiensis* (alignment 573 bp) was 0.2–0.5%, whereas it was 14.3–15% between *N. talmiensis* and *Neotrombicula* sp. A (Table S2) and 23.5–23.8% between *N. talmiensis* and *Neotrombicula* sp. B. Species delimitation grouped three single haplotype COI sequences within one OTU.

The null distance was recorded between five 28S sequences (alignment 734 bp).

#### *Neotrombicula vulgaris* (Schluger)

*Material examined*. 1 LV (entire specimen mounted on slide): [6] North Macedonia, Kavadarci, 20 Oct. 2020, leg. TP. For metric and meristic data see Table 2.

*Diagnosis* (after Stekolnikov and Daniel 2012): SIF = 7BS-N-3–3111.1000; fPp = B/B/NNB; fCx = 1.1.1; fSt = 2.2; fSc: PL > AM > AL; Ip = 725–916; fD = 4H-(7–10)-(10–15)-(7–16)-(6–11)-…; DS = 43–63; VS = 24–37; NDV = 72–95.

#### Distribution and hosts

Azerbaijan, Bulgaria, Czechia, [?] China, Georgia, Hungary, Iran, Moldova, Poland, Russia, Slovakia, Spain, Turkey, Turkmenistan, Ukraine; hosts: rodents and eulipotyphlas, occasionally bats and humans (Schluger 1955; Gadzhiev and Dubovchenko 1976; Moniuszko and Mąkol 2014; Shamsi et al. 2020, and references therein; Stekolnikov and Mumcuoglu 2022).

#### Remarks

Species new to the fauna of North Macedonia. Identification based exclusively on morphology, with the application of the key provided by Kudryashova (1998). In the only specimen examined, the nine setae were observed in row D on the opisthosoma dorsum, thus the following data should be incorporated in the modified diagnosis of the species: fD = 4H-(7–10)-(9–15)-(….).

#### *Neotrombicula* sp. A

*Material examined*. 2 LV (two exoskeletons mounted on slides; two COI sequences [OQ924409; OQ924412]): [7] Greece, Thasos, 29 Sept. 2021, leg. TP. For metric and meristic data see Table 2.

*Remarks*. The specimens can be identified as *N. talmiensis* based on the key provided by Kudryashova (1998). They share the following character states with *N. talmiensis*: SD < 60; PL > AM = AL; fD = 2H-6–6-(…). The differences between *Neotrombicula* sp. A and *N. talmiensis* pertain to the position of posterolateral setae (PL) in relation to sensilla (S) bases: PL/SB (in *Neotrombicula* sp. A) vs. SB-PL (in *N. talmiensis*), and to the presence of four setae in row E (n = 1) (vs. six setae in *N. talmiensis*). Moreover, some metric character states of one specimen of *Neotrombicula* sp. A, slightly depart from the scope known for *N. talmiensis*, whereas in the case of the second specimen, all character states fall within the range recognized for *N. talmiensis* (see also Table 2).

Two single haplotype COI sequences obtained from two specimens assigned to *Neotrombicula* sp. A represented one OTU and the genetic distance between them (alignment 573 bp) was 0.7%, whereas it was 14.3–15% between *Neotrombicula* sp. A and *N. talmiensis* (Table S2). The results confirm a separate identity of *Neotrombicula* sp. A; however, the ultimate decision on species affiliation (assignment to already known species or providing with a new name, i.e., the description of a new species) should be made after examination of more extensive material.

#### *Neotrombicula* sp. B

*Material examined*. 1 LV (exoskeleton lost; one COI [OQ924408] and one 28S [OQ925890] sequence): [4] Albania, Perlat, 10 Oct. 2020, leg. TP.

*Remarks*. In the lack of exoskeleton, the identity of *Neotrombicula* sp. B could be assessed based exclusively on molecular data. Species delimitation allowed to assign [OQ924408] to yet separate OTU. The genetic distance between the COI sequence of the only specimen of *Neotrombicula* sp. B and *N. talmiensis* (alignment 573 bp) was 23.5–23.8%, and 23.7–23.9% between *Neotrombicula* sp. B and *Neotrombicula* sp. A (Table S2).

A comparison between 28S single haplotype sequences of [OQ925890] and *N. talmiensis* [OQ925891] (alignment 734 bp) revealed the distance of 2.2%, which also votes for separate species identity of *Neotrombicula* sp. A.

### *Brunehaldia*

*Diagnosis* (after Stekolnikov and Daniel 2012). SIF = 7BS-B-3–211(0)1(0)0.0000. fCx = 1.1.(1–5). Eyes absent. Scutum trapezoidal, with rounded or concave posterior margin; sensillary bases situated posterior to the level of PL; AL and PL approximate to each other. Sensilla clavate, fusiform or pyriform, covered with setules. Two or more pairs of humeral setae. Scutal and idiosomal setae covered with long thin barbs.

*Distribution and hosts*. Afghanistan, Azerbaijan, Bulgaria, Egypt, France (Corsica), Israel, Iran, Kazakhstan, Kosovo, Kyrgyzstan, Morocco, North Macedonia, Pakistan, Spain, Ukraine, Uzbekistan, Russia, Turkey, Turkmenistan; parasites of rodents and of eulipotyphlas, rarely found on birds (Shamsi et al. 2020, and references therein; Stekolnikov and Daniel 2012).

#### *Brunehaldia* sp.

*Material examined*. 1 LV (exoskeleton mounted on slide): [8] Greece, Pylos, 9 Oct. 2021, leg. TP. For metric and meristic data see Table 3.

**Table 3:** Morphological data on *Brunehaldia bulgarica, Brunehaldia* sp., and *Hirsutiella zachvatkini*

Trait	*Brunehaldia bulgarica*			*Brunehaldia* sp.	*Hirsutiella zachvatkini*				
	Vercammen-Grandjean and Kolebinova 1966 Bulgaria^a^	Imaz et al. 2006 Spain (n = 9)	Stekolnikov and Daniel 2012 Turkey (n = 10)	This study Greece (n = 1)	Stekolnikov 2001^b^ GER, CZE, SLK, MOL, RUS (n = 41)	Imaz et al. 2006 Spain (n = 11)	Moniuszko et al. 2015 Poland (n = 133)	This study Albania (n = 1)	This study North Macedonia (n = 1)
SB	27	23–28	21–23	**15**	29–37	31–38	30–40	35	35
AW	57	55–60	53–60	**48**	70–82	73–83	60–98	72	71
PW	66	68–73	54–65	**52**	78–95	93–103	76–100	84	83
AP	15	15–18	13–14	**11**	24–33	30–38	20–34	25	25
ASB	27	28–33	24–29	**16**	41–48	39–45	26–44	43	41
PSB	11	9–13	11–14	11	14–19	15–18	12–26	17	14
P-PL	–	20–25	22–25	**19**	25–34	–	18–33	32	27
AM	24	23–28	18–24	18	47–60	50–63	41–59	48	53
AL	37	30–38	31–37	**20**	45–63	60–68	41–61	47	50
PL	48	43–50	41–47	**35**	67–87	88–106	60–85	60	69
S	33	28	23–29	**19**	86–108	88–100	81–131	–	86
SD	38	38–43	38–42	**27**	57–66	54–60	40–63	60	55
Leg I	179	210–233	230–256	196	362–382	337–386	309–460	318	298
Leg II	205	200–208	198–211	**150**	301–355	327–356	284–409	297	259
Leg III	596	243–250	234–252	**191**	342–391	256–396	316–470	343	306
Ip	596	665–685	669–712	**511**	997–1120	1020–1119	914–1325	958	864
D_min_	36	30–45	22–27	**21**	41–63	65–75	51–60	48	–
D_max_	41	45–50	44–49	**36**	58–77	80–90	61–69	60	–
V_min_	20	20–33	17–21	**15**	29–53	33–60	22–37	25	–
V_max_	33	25–38	32–35	**24**	38–68	45–78	51–58	53	–
H	36–42	43–48	37–43	–	59–85	93–100	67–79	64	–
DS	58	55–61	51–58	58	73–98	68–95	82–96	> 87	–
VS	62	60–64	47–59	48	56–91	64–112	52–72	–	–
NDV	120	115–124	101–115	106	145–189	150–185	136–166	147	140

Values of metric traits of *Brunehaldia* sp. departing from the scope of variation of *B. bulgarica* are indicated in bold. Trait codes (first column) explained in Stekolnikov (2013)

^a^No data on the sample size

^b^Material originating from Germany, Czechia, Slovakia, Moldova, Russia

*Remarks*. New country record (Greece) and new host record. The only specimen examined displays the SIF formula 7BS-B-3-2111.0000. Comparison of the material with the data for eight hitherto described members of *Brunehaldia* points to the closest similarity of our specimen to *Brunehaldia bulgarica* (Vercammen-Grandjean et Kolebinova). The following character states fall within the range of diagnostic traits of *B. bulgarica*: fPp = B/B/BBB; fCx = 1.1.3; fSt = 2.2; NDV = 106; DS = 58; VS = 48. However, we could observe also the smaller scutum in the specimen examined, as well as some other departing character states: SB = 15, Ip = 511, fD = 4H-10–12-10-(…), PL > AL ≥ AM vs. SB = 21–23, Ip = 669–712, fD = 4H-8-(10–12)-(10–13)-(9–11)-6–2 and PL > AL > AM for *B*. *bulgarica*.

Due to the number and significance of differences, and in view of the lack of molecular data that would allow further comparison, we have refrained from a final decision on the affiliation of the specimen, until more material is available.

### *Hirsutiella*

#### *Hirsutiella zachvatkini* (Schluger)

*Material examined*. 2 LV (one entire specimen and one exoskeleton, mounted on slides): [5] Albania, Langarica Canyon, 15 Oct. 2020, leg. TP; [6] North Macedonia, Kavadarci, 20 Oct. 2020, leg. TP. For metric and meristic data see Table 3.

*Diagnosis* (after Stekolnikov 2001). SIF = 7BS-B-3–2111.1000; fPp = B/B/BBB; fSt = 2.2; fSc: PL > AL > AM or AM > AL; Ip = 997–1120; DS = 86; VS = 73; NDV = 159.

*Distribution and hosts*. Albania, Austria, Azerbaijan, Belarus, Bulgaria, Czechia, France, Georgia, Germany, Hungary, Kazakhstan, Kyrgyzstan, Latvia, North Macedonia, Moldova, Poland, Romania, Russia, Slovakia, Slovenia, Spain, Sweden, Switzerland, Ukraine, former Yugoslavia; hosts: rodents and eulipotyphlas (Moniuszko and Mąkol 2014, and references therein).

#### Remarks

Identification was based exclusively on morphology. In general, the values of morphometric traits fell within the range of *H. zachvatkini* or were slightly lower than data provided for the species by Stekolnikov (2001) and Moniuszko et al. (2015). In relation to most characters, they were also slightly lower than data provided by Imaz et al. (2006). The value that more markedly departed from species diagnosis pertained to *index pedibus* (Ip = 864) recorded for the specimen from Albania.

Stekolnikov (2001), in the diagnosis of *N. zachvatkini* referred to exact metric and meristic character states, except for Ip. The diagnosis, however, was not modified despite the relatively wide range of morphometric data recorded for specimens assigned to the species (Table 3), reported by various authors. The latter may be due to the uncertain status of *H. zachvatkini*, being likely to represent a species complex, thus should be verified based on more extensive material from the geographic range of ‘*H. zachvatkini*’, supported by molecular evidence.

### *Schoutedenichia*

*Diagnosis* (after Stekolnikov 2018). SIF = 4B(4BS, 5B)-N(B)-3-2(1)1(0)1(0)0.0000. Eyes 2 + 2 or 1 + 1. Scutum trapezoidal, with straight or concave posterior margin; sensillary bases situated far apart (distance between sensilla larger than distance between sensillum and lateral scutal margin). Sensilla clavate to globose, covered with setules.

#### Distribution and hosts

Palearctic, Nearctic, as well as Afrotropical and Australian regions; hosts: rodents, eulipotyphlas, squamates (Stekolnikov and Daniel 2012; Moniuszko and Mąkol 2014; Stekolnikov 2018).

#### Remarks

The genus comprises six subgenera, and 104 nominal species (Stekolnikov 2019; Nielsen et al. 2021; Stekolnikov and Matthee 2022). We refrained from ascertaining the systematic affiliation of the material obtained during the present study to the particular subgenus due to the unstable criteria of recognition of subgenera distinguished within *Schoutedenichia*.

#### *Schoutedenichia* sp. A

*Material examined*. 1 LV (exoskeleton mounted on slide): [6] North Macedonia, Kavadarci 20 Oct. 2020, leg. TP. For metric and meristic data see Table 4.

**Table 4:** Morphological data on *Schoutedenichia* spp.

Trait	*Schoutedenichia* (*Platytrichia*) *krampitzi*				*Schoutedenichia* (*Pentachia*) *xeri*	*Schoutedenichia* sp. A	*Schoutedenichia* sp. B
	Willmann 1955 Italy^a^	Kolebinova 1992 Bulgaria^a^	Imaz et al. 2006 Spain (n = 10)	Stekolnikov and Daniel 2012^b^ Russia (n = 10)	Taufflieb 1966 Central African Republic (n = 10)	This study North Macedonia (n = 1)	This study Albania (n = 1)
SB	40	34–45	33–40	38–49	44	45	37
AW	60	55–68	58–63	63–68	55	69	56
PW	80	76–90	75–90	83–89	72	93	72
AP	40	38–56	38–43	37–41	36	46	37
ASB	27	29–34	25–30	29–32	26	32	24
PSB	20	21–25	20–23	21–23	24	25	24
P-PL	–	–	–	9–14	–	14	10
AM	40	38–43	35–43	38–41	28	38	41
AL	47	38–45	35–43	42–48	28	50	30
PL	47	38–52	48–53	48–54	43	53	36
S	34	34–43	38	33	–	–	–
SD	47	50–56	45–53	50–56	50	57	48
Leg I	–	215–265	208–248	261–293	256	233	267
Leg II	–	195–238	208–228	236–257	216	207	209
Leg III	–	238–275	238–267	279–293	253	258	250
Ip	–	648–778	663–743	794–833	725	699	727
D_min_	–	30/38	30–38	31–33	–	30	–
D_max_	–	38/45	45	43–47	–	44	–
V_min_	–	21/29	23–30	23	–	28	–
V_max_	–	28/42	25–38	40	–	32	–
H	–	40–50	48–53	47–54	–	50/52	–
DS	64	64	41–52	60–74	30–40	–	–
VS	–	72	54–67	53–68	20–34	–	–
NDV	–	136	103–113	116–140	128	120	> 88

Trait codes (first column) explained in Stekolnikov (2013)

^a^No data on the sample size

^b^With the following exceptions: S (n = 1); V_min_ and V_max_ (n = 3)

*Remarks*. The specimen revealed similarity to *Schoutedenichia* (*Platytrichia*) *krampitzi* (Willmann) (e.g., SIF = 4B-B-3-1110.0000; fCx = 1.1.1.; for comparison of metric and meristic data of *S. krampitzi* and *Schoutedenichia* sp. A, see Table 4) and differed from the latter species in the presence of three barbed setae on palpal tibia [vs. two nude setae and one barbed seta in* S.* (*P.*) *krampitzi*]. The observed difference may contribute to the intraspecific variation of *S.* (*P.*) *krampitzi*; however, the further inference should be based on a larger series of specimens.

#### *Schoutedenichia* sp. B

*Material examined*. 1 LV (exoskeleton mounted on slide; COI sequence [OQ924405]): [5] Albania, Langarica Canyon, 15 Oct. 2020, leg. TP. For metric and meristic data see Table 4.

*Remarks*. The SIF formula in the examined specimen (5B-B-3-2110.0000) pointed to its affiliation with *Schoutedenichia* (*Pentachia*); however, the palpal setal formula in relation to palp femur, palp genu and palp tibia (B/N/BBN) differed from the one observed in the only species, *Schoutedenichia* (*Pentachia*) *xeri* Taufflieb, known for the subgenus (B/B/BBB). Other differences, whose importance should be confirmed in future, pertained to the shape of sensilla bases on scutum [dorsal and ventral cuticular frames present in our specimen; only dorsal frame around the sensilla base—in *S.* (*P*.) *xeri*], the serration of chelicera [smooth cheliceral blade in our specimen; cheliceral blades serrated dorsally in *S.* (*P*.) *xeri*] and number of setae in the row C on opisthosoma dorsum [2H-10-(…) in our specimen, 2H-6-(…) in *S.* (*P*.) *xeri*]. A comparison of metric and meristic data of *S.* (*P*.) *xeri* and *Schoutedenichia* sp. B is provided in Table 4. Due to the observed differences, *S.* (*P*). *xeri* and the specimen examined in the present study may represent two distinct species. The latter should be confirmed, however, through inference based on more extensive material.

The genetic distance between the COI sequence of *Schoutedenichia* sp. B, and *Schoutedenichia* (*S.*) *centralkwangtunga* (Mo, Chen, Ho et Li) (KY971498), being the only sequence of *Schoutedenichia* available in the GenBank, equals 20.4%.

### Multiple invasions

We observed four cases of synchronous and syntopic parasitism of chiggers on the edible dormouse, and all of them pertained to the representatives of different genera that entered the pair-wise interaction. Larvae of *Leptotrombidium* sp. A and of *Neotrombicula* sp. B were parasitising the same host specimen, a juvenile male of *G. glis* in Albania. The presence of *Schoutedenichia* sp. B and *H. zachvatkini* on the same host (adult female) was stated also in Albania. The other cases of multiple parasitism pertained to *N. talmiensis* and *Brunehaldia* sp. (on adult female) in Greece, and to *N. vulgaris* and *Schoutedenichia* sp. A (on juvenile female) in North Macedonia.

### Phylogenetic relationships

The support for species delineated based on molecular and morphological criteria was tested on 417 bp dataset consisting of 114 COI sequences (12 obtained during present study, and 100 assigned to chiggers and two of outgroup taxa, retrieved from the GenBank).

**Fig. 4:**
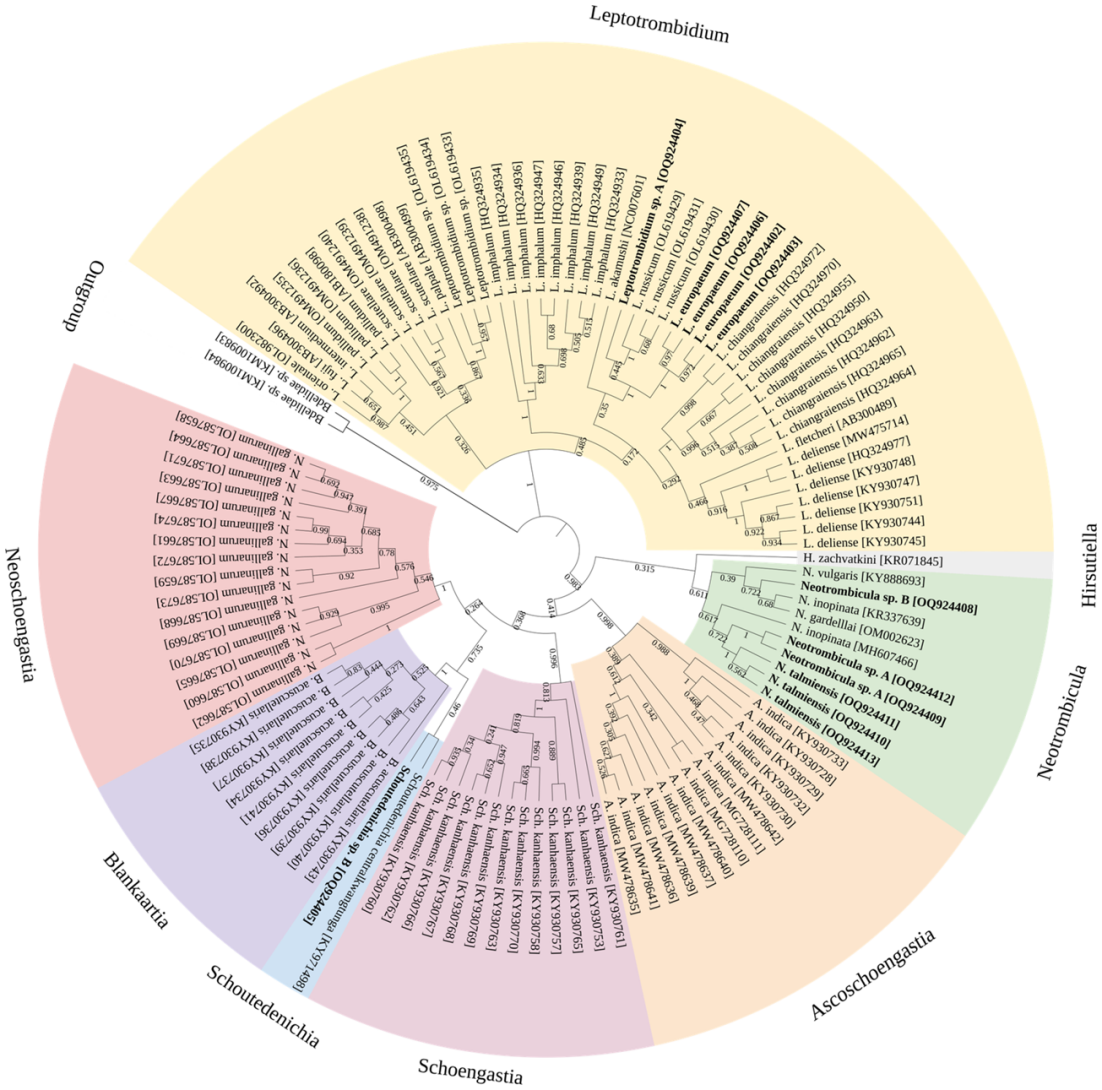
Maximum Likelihood (ML) tree based on COI dataset with bootstrap values on branches

Results confirmed the clear separation of genera. Sequences of Trombiculidae collected from *G. glis* were clearly delineated and scattered between distinct clades containing *Leptotrombidium*, *Neotrombicula* and *Schoutedenichia* (Fig. 4). The corresponding branches were either well- or a strongly supported.

## Discussion

The representatives of five genera and 10 species of Trombiculidae were recognized in the material collected from *G. glis*. The successful amplification and sequencing of COI was carried out for three genera and six species. For the remaining taxa the molecular analyses failed which confirms the low success rate of obtaining COI sequences for chiggers (e.g., Antonovskaia 2018; Bassini-Silva et al. 2018; Kumlert et al. 2018).

The study shows a discrepancy between the results of molecular and morphological identification. The scale of this discrepancy cannot be estimated at present due to the scarcity of sequences that allow the comparison. The scope of intraspecific variation of morphological traits largely varies across Trombiculidae and seems to be related to the frequency of records of morphologically distinguished species. Only sometimes the latter can be translated into greater geographic distribution of the species. In the case of *L. europaeum*, we could establish the wider, than hitherto observed, variation in the number of setae assigned to the row E on the opisthosoma dorsum, the lower values of Ip, and several other metric data that contribute to wider variation known for the species (Table 1), as reported by various authors. Only the indistinct differences recorded between *L. europaeum* and *Leptotrombidium* sp. A (value of PW and DS), could contribute to widening the intraspecific variation as well, whereas all other character states recognized for two specimens of *Leptotrombidium* sp. A fell within the variability range of *L. europaeum* reported in the present study. The measure of genetic distance between these two species (10.1–11.5%) voted for their separate identity. In the lack of clear morphological differences between them, we would attribute the phenomenon to hidden biodiversity of chiggers, even more difficult to detect due to the likely presence of relatively wide host spectra of ectoparasitic larvae, and the already confirmed (Moniuszko et al. 2015) phenomenon of phenotypic plasticity expressed in host-associated differences in morphometric traits of parasitic larvae.

As in *L. europaeum*, the wider than hitherto observed range of metric and meristic traits, at high level of identity of COI sequences, was recognized in two (out of eight) specimens assigned to *N. talmiensis*. The separate identity of *N. talmiensis*, *Neotrombicula* sp. A and *Neotrombicula* sp. B was confirmed by the measure of genetic distance (see also Table S2). The morphological differences between *N. talmiensis* and *Neotrombicula* sp. A (in the lack of voucher of *Neotrombicula* sp. B) were small, and pertained to slight departure of data of only one specimen from the range specified for *N. talmiensis*. Nevertheless, the concept of *N. talmiensis* as a group of closely related species (Stekolnikov 2001, 2002; Shatrov and Antonovskaia 2021) cannot be re-appraised without application of molecular tools.

The re-assessment of the usefulness of characters in species description should involve both qualitative and quantitative traits. Among the former ones, the shape of galeala and the structure of setae on palps—with special reference to palp genu, tibia and tarsus—should be prioritized. As we do not know the actual variation of morphological traits at the intraspecific level, the application of morphological keys in species identification is of limited value and should be done with extreme care.

The obtained results indicate the likely occurrence of cryptic species within Trombiculidae, and they indicate that the set of morphological traits used to distinguish species must be verified due to the high variability observed at the intraspecific level. Such verification is necessary because of the growing interest in the group, resulting also from the medical-veterinary importance of these mites and entailing the risk of an increasing number of misidentifications.

The recognition of species boundaries within Trombiculidae, based almost exclusively on morphological criteria, is in a state of flux. The species affiliation of all specimens for which we failed to obtain COI sequences, would remain provisional until more extensive material can be examined and verified with the application of morphological as well as molecular methods (including multiple markers). Thus, we fully support the opinion of Kumlert et al. (2018) who claim that intraspecific variation of morphometric characteristics supported by molecular genotyping needs to be more comprehensively investigated for the members of the Trombiculidae.

The molecular species delimitation could also be helpful in inferring on species diversity. The method—to our best knowledge applied for the first time in relation to trombiculid mites, although tested already for other Parasitengona groups (e.g., Stålstedt et al. 2016; Blattner et al. 2019; Tyukosova et al. 2022)—allowed us to hypothesize on actual species borders and to juxtapose it with the results of morphological identification.

The distance methods were confirmed by means of phylogenetic analyses. Resolving discrepancies related to the position of species (with sequences retrieved from the GenBank) in the phylogenetic tree (e.g., *N. inopinata*) is beyond the scope of the present study and should be clarified in a separate project.

Multiple invasions, quite commonly observed in Trombiculidae (e.g., Moniuszko et al. 2018; Jacinavicius et al. 2021; Stekolnikov et al. 2022), have also been discovered in the present study. The likely occurrence of simultaneous parasitism of representatives of various genera and species on the same host, entails the necessity to examine all specimens which are present in the material. The inference on species composition cannot be made without in-depth morphological examination, the best supported by molecular analyses.

## Supplementary Information

Below is the link to the electronic supplementary material.

Supplementary file1 (DOCX 29 kb)
