# Supplementary material for: Let me know your name: a study of chigger mites (Acariformes: Trombiculidae) associated with the edible dormouse (Glis glis) in the Carpathian–Balkan distribution gradient
Source: Exp Appl Acarol. 2023 Aug 8;91(1):1–27. doi: 10.1007/s10493-023-00824-0 (PMC10462554; doi:10.1007/s10493-023-00824-0)
Supplement: Supplementary file 1 — Supplementary file1 (DOCX 29 kb) [file 10493_2023_824_MOESM1_ESM.docx]

**Table S1** List of taxa and corresponding COI and 28S sequences retrieved from GenBank and applied in delimitation methods (rows 4–19) and in the phylogenetic analysis (rows 1–24)

| Item no. | Species | COI | 28S rDNA | Country (collection site) | References |
| --- | --- | --- | --- | --- | --- |
| 1. | *Ascoschoengastia indica* (Hirst, 1915) | MW478635  MW478636  MW478637  MW478639  MW478640  MW478641  MW478642 | -  -  -  -  -  -  - | Thailand | Wulandhari et al. (2021) |
|  |  | MG728110  MG728111 | -  - | Thailand | Kumlert et al. (2018) |
|  |  | KY930728  KY930729  KY930730  KY930732  KY930733 | -  -  -  -  - | Laos | Kumlert et al. (2018) |
| 2. | *Blankaartia acuscutellaris* (Walch, 1922) | KY930734  KY930735  KY930736  KY930737  KY930738  KY930739  KY930740  KY930741  KY930743 | -  -  -  -  -  -  -  -  - | Laos | Kumlert et al. (2018) |
| 3. | *Hirsutiella zachvatkini* (Schluger, 1948) | KR071845 | - | Poland | Moniuszko et al. (2015) |
| 4. | *Leptotrombidium akamushi* (Brumpt, 1910) | NC007601 | - | Japan | Mitani et al. [2005]^2^ |
| 5. | *Leptotrombidium chiangraiensis* Tanskul *et* Linthicum, 1997^6^ | HQ324950  HQ324955  HQ324962  HQ324963  HQ324964  HQ324965  HQ324970  HQ324972 | -  -  -  -  -  -  -  - | Thailand | Takhampunya et al. [2016]^3^ |
| 6. | *Leptotrombidium* (*Leptotrombidium*) *deliense* (Walch, 1922) | KY930744  KY930745  KY930747  KY930748  KY930751 | -  -  -  -  - | Laos | Kumlert et al. 2018 |
|  |  | MW475714 | - | Thailand | Wulandhari et al. [2021]^4^ |
|  |  | HQ324977 | - | - | Takhampunya et al. [2016]^3^ |
| 7. | *Leptotrombidium fletcheri* (Womersley *et* Heaslip, 1943) | AB300489 | - | no data | Mitani et al. [2016]^5^ |
| 8. | *Leptotrombidium fuji* (Kuwata, Berge *et* Philip, 1950) | AB300496 | - | no data | Mitani et al. [2016]^5^ |
| 9. | *Leptotrombidium imphalum* Vercammen-Grandjean *et* Langston, 1976 | HQ324933  HQ324934  HQ324935  HQ324936  HQ324939  HQ324946  HQ324947  HQ324949 | -  -  -  -  -  -  -  - | Thailand | Takhampunya et al. [2016]^3^ |
| 10. | *Leptotrombidium intermedium* (Nagayo, Mitamura *et* Tamiya, 1920) | AB300492 | - | no data | Mitani et al. [2016]^5^ |
| 11. | *Leptotrombidium orientale* (Schluger, 1948) | OL982300 | - | South Korea | Lee H.S. [2021]^1^ |
| 12. | *Leptotrombidium pallidum* (Nagayo, Miyagawa, Mitamura *et* Tamiya, 1919) | OM491235  OM491236 | -  - | South Korea | Lee H.S. [2022]^1^ |
|  |  | AB180098 | - | Japan | Shao et al. (2005) |
| 13. | *Leptotrombidium palpale* (Nagayo, Miyagawa, Mitamura *et* Tamiya, 1919) | *AB300499 | - | no data | Mitani et al. [2016]^5^ |
| 14. | *Leptotrombidium russicum* (Oudemans, 1903) | OL619429  OL619430  OL619431 | -  -  - | Poland | Zajkowska and Mąkol (2022) |
| 15. | *Leptotrombidium scutellare* (Nagayo, Miyagawa, Mitamura *et* Tenjin, 1921) | AB300498 | - | no data | Mitani et al. [2016]^5^ |
|  |  | OM491238  OM491239  OM491240 | -  -  - | South Korea | Lee H.S. [2022]^1^ |
| 16. | *Leptotrombidium* sp. | OL619433  OL619434  OL619435 | ~~-~~  -  - | Poland | Zajkowska and Mąkol (2022) |
| 17. | *Neotrombicula gardellai* (Kardos, 1961) | OM002623 | OM108508 | South Korea | Lee H.S. [2021]^1^ |
| 18. | *Neotrombicula inopinata* (Oudemans, 1909) | MH607466 | - | Poland | Moniuszko et al. (2018) |
|  |  | KR337639 | - | Spain | Santibáñez-Sáenz (2015) |
| 19. | *Neotrombicula vulgaris* (Schluger, 1955) [PAL] | KY888693 | - | Poland | Moniuszko et al. (2017) |
| 20. | *Neoschoengastia gallinarum* (Hatori, 1920) | OL587658  OL587659  OL587660  OL587661  OL587662  OL587663  OL587664  OL587665  OL587667  OL587668  OL587669  OL587670  OL587671  OL587672  OL587673  OL587674 | -  -  -  -  -  -  -  -  -  -  -  -  -  -  -  - | China | Tao et al. (2022) |
|  |  | -  -  -  - | MK418680  MK418681  MK418682  MK418683 | China | Zhou et al. (2020) |
| 21. | *Schoutedenichia* (*Schoutedenichia*) *centralkwangtunga* (Mo, Chen, Ho *et* Li, 1959) | KY971498 | - | Laos | Kumlert et al. (2018) |
| 22. | *Schoengastia* (*Schoengastia*) *kanhaensis* Mitchell *et* Nadchatram, 1966 | KY930753  KY930757  KY930758  KY930760  KY930761  KY930762  KY930763  KY930765  KY930766  KY930767  KY930768  KY930769  KY930770 | -  -  -  -  -  -  -  -  -  -  -  -  - | Laos | Kumlert et al. (2018) |
| 23. | Bdellidae sp. 1 | KM100983 | - | no data | Dabert et al. (2016) |
| 24. | Bdellidae sp. 2 | KM100984 | - | no data | Dabert et al. (2016) |

^1-5^ sequences retrieved from GenBank, that have not appeared in journal publications (year of submission – in square brackets): ^1^ Lee H.S. [2021]; ^2^ Mitani H., Shao R., Takahashi M., Fukunaga M. [2005]; ^3^ Takhampunya R., Korkusol A., Jaichapor B., Ponlawat A., Monkanna T., Khlaimanee N., Richardson J.H., Evans B.P. [2016]; ^4^ Wulandhari S.A., Paladsing Y., Saesim W., Charoennitiwat V., Sonthayanon P., Kumlert R., Morand S., Sumruayphol S., Chaisiri K. [2021]; ^5^ Mitani H., Yuasa S., Takahashi M., Fukunaga M. [2016]; ^6^ species synonymized with *Leptotrombidium imphalum* Vercammen-Grandjean *et* Langston, 1976 by Stekolnikov (2013); the synonymization not followed by Kumlert et al. (2018)

**Table S2** The K2P distance (%) of COI sequences (alignment 573 bp) received from chiggers collected from *Glis glis*

|  | | OQ924402 | OQ924403 | OQ924407 | OQ924406 | OQ924404 | OQ924408 | OQ924413 | OQ924411 | OQ924410 | OQ924409 | OQ924412 |
| --- | --- | --- | --- | --- | --- | --- | --- | --- | --- | --- | --- | --- |
| *Leptotrombidium europaeum* | OQ924402 |  |  |  |  |  |  |  |  |  |  |  |
|  | OQ924403 | 1.2 |  |  |  |  |  |  |  |  |  |  |
|  | OQ924407 | 2.7 | 2.9 |  |  |  |  |  |  |  |  |  |
|  | OQ924406 | 2.5 | 2.7 | 0.9 |  |  |  |  |  |  |  |  |
| *Leptotrombidium* sp. A | OQ924404 | 10.1 | 10.8 | 11.0 | 11.5 |  |  |  |  |  |  |  |
| *Neotrombicula* sp. B | OQ924408 | 36.7 | 36.7 | 36.4 | 35.8 | 37.1 |  |  |  |  |  |  |
| *Neotrombicula talmiensis* | OQ924413 | 35.8 | 36.1 | 36.4 | 36.1 | 37.5 | 23.5 |  |  |  |  |  |
|  | OQ924411 | 35.8 | 36.1 | 36.4 | 36.1 | 37.5 | 23.5 | 0.3 |  |  |  |  |
|  | OQ924410 | 35.5 | 35.8 | 36.1 | 35.8 | 37.2 | 23.8 | 0.2 | 0.5 |  |  |  |
| *Neotrombicula* sp. A | OQ924409 | 36.0 | 36.9 | 36.4 | 36.4 | 36.8 | 23.9 | 14.6 | 15.0 | 14.3 |  |  |
|  | OQ924412 | 36.3 | 37.2 | 36.6 | 36.6 | 36.5 | 23.7 | 14.6 | 15.0 | 14.3 | 0.7 |  |
| *Schoutedenichia* sp. B | OQ924405 | 40.5 | 41.8 | 43.6 | 42.6 | 40.6 | 32.1 | 30.5 | 31.1 | 30.2 | 28.4 | 29.0 |
